# Diosmin mitigates high glucose-induced endoplasmic reticulum stress through PI3K/AKT pathway in HK-2 cells

**DOI:** 10.1186/s12906-022-03597-y

**Published:** 2022-04-27

**Authors:** Jiuhong Deng, Chao Zheng, Zhou Hua, Haideng Ci, Guiying Wang, Lijing Chen

**Affiliations:** 1 Wenzhou Medical University, Chashan Higher Education Park, Wenzhou City, 325035 Zhejiang Province China; 2 Department of Endocrinology, Second People’s Hospital of Pingyang County, Wenzhou City, 325405 Zhejiang Province China; 3 The Second Affiliated Hospital Zhejiang University, School of Medicine, Jiefang Road 88, Shangcheng District, Hangzhou City, 310009 Zhejiang Province China; 4 Department of Nephrology, The Poeple’ s Hospital of Suichang County, Lishui City, 323300 Zhejiang Province China; 5 Department of Endocrinology and Nephrology, Jiande Hospital of Traditional Chinese Medicine, Hangzhou City, 311600 Zhejiang Province China; 6 Shangyu People’s Hospital of Shaoxing, Shaoxing City, 312300 Zhejiang Province China; 7 Department of Nephrology, Huzhou Central Hospital; Affiliated Central Hospital of Huzhou University; Affiliated Huzhou Hospital; Zhejiang University School of Medicine, Huzhou City, 313000 Zhejiang Province China

**Keywords:** Diabetic nephropathy, Diosmin, High glucose, Endoplasmic reticulum stress, Phosphatidylinositol 3-kinase/protein kinase-B pathway

## Abstract

**Background:**

Diosmin has been reported to treat diabetes, but its role in diabetic nephropathy (DN) remains unclear. This research investigated the mechanism by which diosmin alleviated high glucose (HG)-induced HK-2 cell injury.

**Methods:**

First, we used CCK-8 to detect the effect of 0.1, 1, or 10 μg/mL diosmin on the viability of HK-2 cells treated with normal glucose or HG. Next, we used flow cytometry, automatic biochemical analyzer, ELISA, immunofluorescence, and colorimetric assay kit to examine the apoptosis, oxidative stress, inflammatory factors, and Caspase-3 expression in HK-2 cells. Thereafter, we used the western blot and qRT-PCR to examine the expression of the endoplasmic reticulum stress-, oxidative stress-, inflammation-, apoptosis-, and autophagy, and PI3K/AKT pathway-related factors.

**Results:**

Diosmin was non-cytotoxic to normal HK-2 cells and enhanced the HK-2 cell viability suppressed by HG. Meanwhile, diosmin restrained apoptosis, the contents of MDA, pro-inflammatory factors, and Caspase-3 but intensified the contents of SOD and CAT induced by HG. We further confirmed that diosmin blunted oxidative stress-, inflammation-, apoptosis-, and autophagy-related factors expression induced by HG via restraining the CHOP and GRP78 expressions. Further, we also discovered that PTEN level was restrained and the ratios of p-PI3K/PI3K and p-AKT/AKT were enhanced in HK-2 cells induced by HG, which was reversed by co-treatment of HG and diosmin.

**Conclusions:**

Our study manifested that diosmin alleviated the HG-mediated endoplasmic reticulum stress injury in HK-2 cells via restraining the PI3K/AKT pathway.

**Supplementary Information:**

The online version contains supplementary material available at 10.1186/s12906-022-03597-y.

## Introduction

Diabetic nephropathy (DN) is one of the manifestations of systemic microangiopathy in diabetes mellitus (DM), and about 40% of DM patients suffer from different degrees of chronic kidney disease [1]. DN is characterized by nephritis, urinary albumin, decreased glomerular filtration rate, glomerulosclerosis, and renal interstitial fibrosis, which are the main causes of end-stage renal disease [2–4]. The pathogenesis of DN is complex, and there are many causative agents, such as abnormal hemodynamics, oxidative stress, inflammatory response, glucose metabolism disorder, and so on [5, 6], but a single hypothesis cannot fully explain the systemic pathogenesis of DN. Therefore, exploring the mechanism that mediates DN is very important for the prevention and treatment of DN.

In recent years, natural originated compounds have shown the advantages of multiple targets, multiple links, and few side effects in the prevention and treatment of DN. Among them, diosmin is a micro powdered and purified flavonoid, which is extracted from citrus [7]. Diosmin is well-known for enhancing venous tension, improving microcirculation, and promoting lymphatic reflux, its main mechanism is to reduce the combination of white blood cells and endothelial cells by down-regulating the inflammatory adhesion molecules, thereby inhibiting inflammation and protecting nerves and blood vessels [8, 9]. More over, some novel potential of diosmin are attracting more and more attention recently. Researchers have found that diosmin has a positive effect on anti-tumor, treatment of diabetes, anti-inflammatory, anti-oxidation, etc. [10]. Urios et al. reported that after 5 months of diosmin treatment in DM rats, the albumin clearance rate was reduced, and the albuminemia was improved [11]. This indicates that diosmin is very useful as adjuvant therapy for the prevention of DN.

In recent years, people’s in-depth research has found that the endoplasmic reticulum stress (ERS) is closely correlated with the initiation and development of DN [12]. And in a high-glucose (HG) environment, the apoptosis of rat renal tubules induced by ERS was observed [13]. The latest research exhibited that inhibition of ERS can improve the levels of urinary albumin, blood creatinine, and blood urea nitrogen in DN rats while reducing the expression of mesangial matrix and podocyte apoptosis [14]. In addition, scholars at home and abroad believed that DN is associated with the excessive activation of PI3K/AKT [15]. The PI3K/AKT pathway can regulate cell proliferation, differentiation, apoptosis, aging, and anti-oxidation [16]. Kasinath et al. found that the activities of PI3K and AKT in the renal cortex of the DN model group are largely enhanced [17]. This showed that ERS and PI3K/AKT pathways play a pivotal role in the pathogenesis of DN. However, it has not been reported whether diosmin alleviates the ERS of HK-2 cells mediated by HG through the PI3K/AKT pathway.

Therefore, in this study, we used human proximal tubular epithelial cells (HK-2) as the experimental model to investigate whether diosmin could attenuate the ERS of HK-2 cells mediated by HG through modulating the PI3K/AKT pathway, thereby exerting a protective effect on renal tubular epithelial cells.

## Methods

### Cell culture

HK-2 (HUCL-013) cells were derived from iCell Bioscience Inc. (China). HK-2 cells were maintained in DMEM (SH30243.01, HyClone, USA) augmented with 10% fetal bovine serum (FBS, 11011-8615, Tianhang, China), and placed in a 37 °C, 5% CO_2_ cell incubator (BB150, Thermo Scientific, USA).

### Cell grouping and treatment

First, to analyze the effect of diosmin on HK-2 cells, HK-2 cells were cultured 5 mmol/L normal glucose or 30 mmol/L HG with or without 0.1, 1, or 10 μg/mL diosmin (61,386, Supelco, USA) for 48 h (h) [18]. Among them, diosmin was completely dissolved in DMSO to produce a 5 mg/mL stock solution. Second, to research the role of diosmin on HG-mediated HK-2 cell injury, the experiments were assigned to the control group (Cells were subjected to 5 mmol/L normal glucose in DMEM for 48 h and the same volume of DMSO for another 48 h), the HG group (Cells were exposed to 30 mmol/L HG in DMEM for 48 h and the same volume of DMSO for another 48 h), and the diosmin / HG + diosmin group (Cells were stimulated with 5 mmol/L normal glucose or 30 mmol/L HG for 48 h and then reacted with 10 μg/mL diosmin for another 48 h).

### Cell counting kit-8 (CCK-8) assay

Trypsin-digested HK-2 cell suspensions (3 × 10^4^ cells/mL) were added into the 96-well plates and then transferred to a cell incubator for culture. After 24 h, the cells were treated based on the above groups. After that, we used the 10 μL of CCK-8 solution (HY-K0301, MCE, USA) to stimulate cells and then placed them in a cell incubator for 3 h. In the end, we used the microplate reader (CMaxPlus, MD, USA) to examine the absorbance (450 nm).

### Apoptosis analysis

The apoptosis was examined using the Annexin V-FITC/propidium iodide (PI) kit (556,547, MD, USA). Cells (1.2 × 10^6^ cells/mL) were subjected to treatment according to the above grouping conditions. After cells were collected, washed, and centrifuged, we used the 1 × binding buffer to adjust cell concentration (1.2 × 10^6^ cells/mL). Then, 5 μL Annexin V-FITC and 10 μL PI were added into each sample tube and then placed at 37 °C away from light for 15 min (min). After each sample tube was added into 1 × binding buffer, we used the flow cytometry (C6, BD, USA) to assess apoptosis.

### Oxidative stress index detection

HK-2 cells (3 × 10^4^ cells/mL) are processed in accordance with the corresponding groupings mentioned above. Subsequently, cells were harvested and centrifuged. Thereafter, we harvested the cell supernatant and used the automatic biochemical analyzer (C16000, ABBOTT LABORATORIES, USA) to assess the content of malonaldehyde (MDA), the activities of superoxide dismutase (SOD), and catalase (CAT).

### Determination of pro-inflammatory cytokines

Human tumor necrosis factor-alpha (TNF-α) kit (BPE10110), human interleukin-1β (IL-1β) kit (BPE10083), and IL-6 kit (BPE10140) were derived from Lengton (Shanghai, China). The prepared cell supernatants were diluted as needed and added to each sample hole along with anti-TNF-α / IL-1β / IL-6 antibody and streptavidin-HRP. Subsequently, each hole was covered with sealing film and gently shaken to mix and then placed at 37 °C for 60 min. After each hole has been thoroughly washed 5 times and patted dry, we added color-developing agents A and B to each well. They were then displayed at 37 °C for 10 min away from the light. Finally, we added the stop solution to each hole, the absorbance (450 nm) was assessed using the microplate reader within 10 min.

### Immunofluorescence assay

We inoculated HK-2 cells (3 × 10^4^ cells/mL) into a 6-well plate with cover glass until the cell confluence reached about 70%. After the cells received different treatments, we fixed the cells with a 4% paraformaldehyde fixation solution (P0099, Beyotime, China) and then rinsed them thoroughly with phosphate buffer (PBS, SH30256.01, Hyclone, USA) for 3 times. After permeating the cell membrane with 0.5% Triton X-100 (T109027, Aladdin, China) in each hole, we sealed the cells with 3% BSA blocking solution (B265993, Aladdin, China) for 30 min. They were then reacted with anti-Caspase-3 antibody (1:500, ab32351, abcam, UK) at 4 °C overnight. After the cells in each well were reacted with goat anti-rabbit IgG H&L (DyLight® 488, 1:500, ab96899, abcam, UK), they were reacted with DAPI (ab104139, abcam, UK). In the end, after mounting, the cover glass was placed under the inverted fluorescence microscope (Ts2-FC, Nikon, Japan) to observe the results.

### Western blot

According to the previous research [19], the proteins in the HK-2 cells were obtained using the RIPA lysis solution (P0013D, Beyotime, China). Their concentrations were examined using the BCA kit (pc0020, Solarbio, China) and then denatured. Next, the proteins were detached by electrophoresis and transferred to a PVDF membrane (10,600,023, GE Healthcare Life, USA). The membrane was then blocked with BSA (4240GR100, BioFroxx, German) for 2 h. Thereafter, the membrane was reacted with the primary antibody at 4 °C overnight, and the next day, they were reacted with the secondary antibody HRP (S0001, Affinity, USA) for 1 h. Finally, the bound antibodies were developed with the ECL reagent (E266188, Aladdin, China) under the chemic luminous instrument (610020-9Q, Clinx, China). The primary antibodies were shown below: anti-caspase 3 antibody (1:2000, AF6311, Affinity, USA), anti-Cleaved caspase 3 antibody (1:2000, AF7022, Affinity, USA), anti-CHOP antibody (1:2000, DF6025, Affinity, USA), anti-GRP78 antibody (1:2000, AF5366, Affinity, USA), anti-MNSOD antibody (1:2000, AF5144, Affinity, USA), anti-NOX2 antibody (1:2000, DF6520, Affinity, USA), anti-IL-1β antibody (1:2000, AF5103, Affinity, USA), anti-IL-18 antibody (1:2000, DF6252, Affinity, USA), anti-NLRP3 antibody (1:2000, DF7438, Affinity, USA), anti-BCL-2 antibody (1:2000, AF6139, Affinity, USA), anti-BAX antibody (1:3000, AF0120, Affinity, USA), anti-BECLIN1 antibody (1:2000, AF5128, Affinity, USA), anti-LC3 antibody (1:1000, AF5402, Affinity, USA), anti-p-PI3K antibody (1:1000, AF3241, Affinity, USA), anti-PI3K antibody (1:1500, AF6241, Affinity, USA), anti-p-AKT antibody (1:1500, AF0016, Affinity, USA), anti-AKT antibody (1:1500, AF6261, Affinity, USA), anti-PTEN antibody (1:1500, AF6351, Affinity, USA), and anti-β-actin antibody (1:15000, AF7018, Affinity, USA). β-actin was used as a housekeeping gene.

### qRT-PCR assay

First, we used Trizol (B511311, Sangon, China) to isolate the total RNA of the HK-2 cells. Next, total RNA was dissolved in DEPC water. Thereafter, we used the universal reverse transcription kit (CW2569, ComWin, China) to construct cDNA. Then the cDNA was amplified by SYBR Premix Ex TaqII (RR820A, Takara, Japan) under a PCR instrument (LightCycler® 96, Roche, Switzerland). The amplification conditions were as follows: pre-denaturation at 95 °C for 10 min, denaturation at 95 °C for 15 s, annealing at 60 °C for 1 min, for a total of 40 cycles. The mRNA level of genes was expressed as 2-ΔΔCt [20]. The primer sequences of the detected genes were shown in Table 1.

**Table 1:** Gene sequence primers

Gene	Forward primer(5′-3′)	Reverse primer(5′-3′)
human CHOP	GGAAACAGAGTGGTCATTCCC	CTGCTTGAGCCGTTCATTCTC
human GRP78	CATCACGCCGTCCTATGTCG	CGTCAAAGACCGTGTTCTCG
human β-actin	CATGTACGTTGCTATCCAGGC	CTCCTTAATGTCACGCACGAT

### Statistical analysis

The statistical analysis was carried out by SPSS software (16.0, IBM, USA). One-way ANOVA analysis followed SNK test was employed for comparing the differences between multiple groups. Kruskal-Wallis H test was used for those with uneven variance. The data were expressed as mean ± standard deviation and a *P*-value of below 0.05 was designated as statistically significant.

## Results

### Effect of diosmin on HK-2 cell vitality

**Fig. 1:**
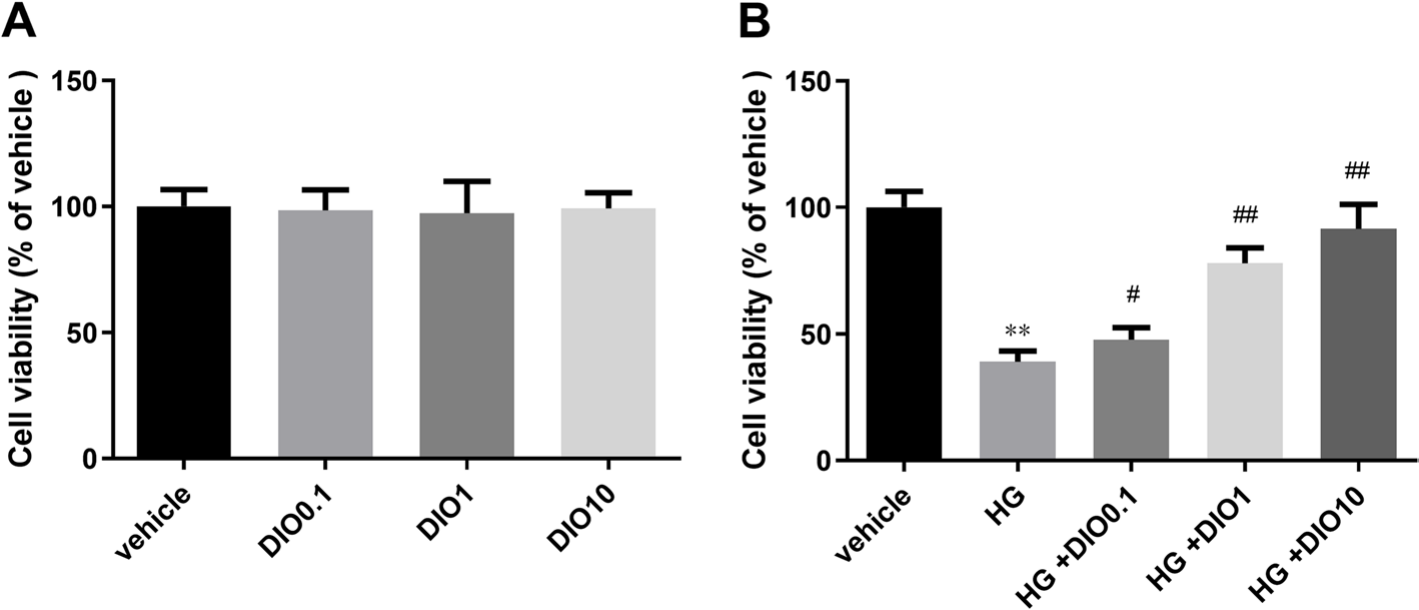
Diosmin has no cytotoxicity and different concentration’ diosmin enhanced the cell vitality induced by high glucose. HK-2 cells cultured with 5 mmol/L normal glucose (**A**) or 30 mmol/L high glucose (**B**) were treated with or without different concentrations of diosmin (0.1, 1, 10 μg/mL) for 48 h. Cell viability was measured by Cell Counting Kit-8 (CCK-8) assay. The results were expressed as the mean ± standard deviation (SD) of three independent experiments. Compared with the vehicle group, ** *p* < 0.01. Compared with the high glucose group, # *p* < 0.05 and ## *p* < 0.01

In this study, we first analyzed the effect of different concentrations of diosmin on HK-2 cells and found that diosmin (0.1, 1, or 10 μg/mL) had no changes on cell viability relative to the vehicle group (Fig. 1A). Next, HK-2 cells cultured with normal glucose or HG were reacted with or without different concentrations of diosmin (0.1, 1, 10 μg/mL). The results manifested that HG evidently restrained the cell vitality than the vehicle group, while diosmin largely elevated the cell vitality in a dose-dependent manner (Fig. 1B, *P* < 0.05). Since 10 μg/mL diosmin obviously elevated cell viability, we chose 10 μg/ mL diosmin for subsequent experiments.

### Diosmin restrained the HG-mediated apoptosis in HK-2 cells

**Fig. 2:**
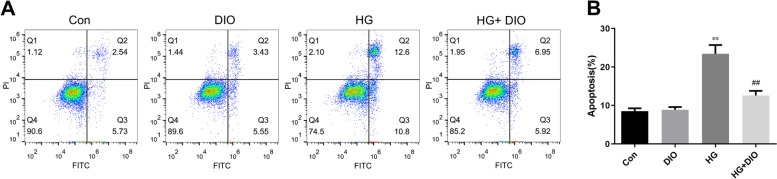
Diosmin restrained the high glucose-mediated apoptosis in HK-2 cells. Effect of diosmin on high glucose-induced apoptosis was determined with Annexin V-FITC/propidium iodide (PI) kit by flow cytometry. **A**. Flow cytometry results with Annexin V-FITC/PI staining. **B**. The ratio of apoptosis among different experiment groups. Apoptosis ratio was early apoptosis percentage plus late apoptosis percentage. Control: Cells were subjected to 5 mmol/L normal glucose in DMEM for 48 h; Diosmin: Cells were subjected to 5 mmol/L normal glucose in DMEM for 48 h and then treated with 10 μg/mL diosmin for another 48 h; High glucose: Cells were exposed to 30 mmol/L high glucose in DMEM for 48 h; High glucose + diosmin: Cells were stimulated with 30 mmol/L high glucose for 48 h and reacted with 10 μg/mL diosmin for another 48 h. The results were expressed as the mean ± standard deviation (SD) of three independent experiments. Compared with the control group, ** *p* < 0.01. Compared with the high glucose group, ## *p* < 0.01

Next, HK-2 cells were treated with diosmin and/or HG. As displayed in Fig. 2A-B, we discovered that diosmin alone without HG did not affect the HK-2 cell apoptosis relative to the control group, while HG stimulation evidently induced the HK-2 cell apoptosis (*P* < 0.01). More importantly, diosmin greatly restrained the HG-mediated apoptosis in HK-2 cells (*P* < 0.01).

### Diosmin decreased the contents of MDA and pro-inflammatory factors but intensified the activities of SOD and CAT in HG-mediated HK-2 cells

**Fig. 3:**
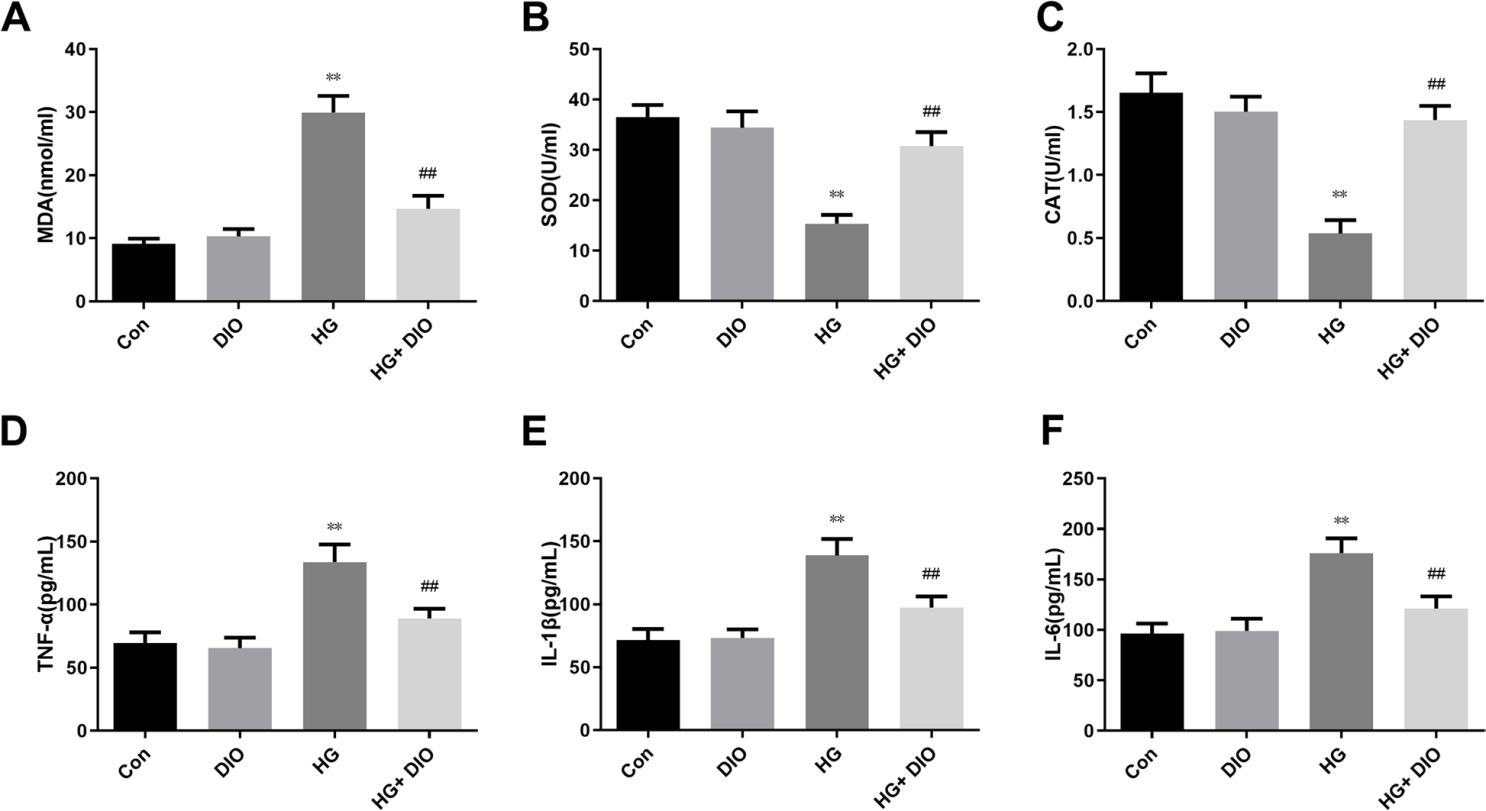
The effect of diosmin on oxidative stress and inflammatory cytokine production in HK-2 cells induced by high glucose. **A-C**. The effect of diosmin on the activities of malonaldehyde (MDA), superoxide dismutase (SOD) and catalase (CAT) was measured by the automatic biochemical analyzer. D-F. The effect of diosmin on the activities of tumor necrosis factor-alpha (TNF-α), interleukin-1β (IL-1β), and IL-6 were measured by enzyme linked immunosorbent assay (ELISA). The results were expressed as the mean ± standard deviation (SD) of three independent experiments. Compared with the control group, * *p* < 0.05 and ** *p* < 0.01. Compared with the high glucose group, ## *p* < 0.01

We analyzed the effect of diosmin on oxidative stress and confirmed that diosmin did not affect MDA content, SOD and CAT activities compared to those in the control group (Fig. 3A-C). Meanwhile, we discovered that a great increase of MDA and a great reduction of SOD and CAT were noticed in the HG-treated alone group than the control group, while diosmin addition in HG-mediated HK-2 cells largely blunted these changes (Fig. 3A-C, *P* < 0.05). Subsequently, we analyzed the effect of diosmin on pro-inflammatory factors, the results showed that the TNF-α, IL-1β, and IL-6 levels in the HG group were greatly intensified than the control group (Fig. 3D-F, *P* < 0.05). Moreover, diosmin extremely weakened the TNF-α, IL-1β, and IL-6 levels induced by HG (Fig. 3D-F, *P* < 0.01).

### The enhancing effect of HG on the expression of Caspase-3 was mitigated by diosmin intervention

**Fig. 4:**
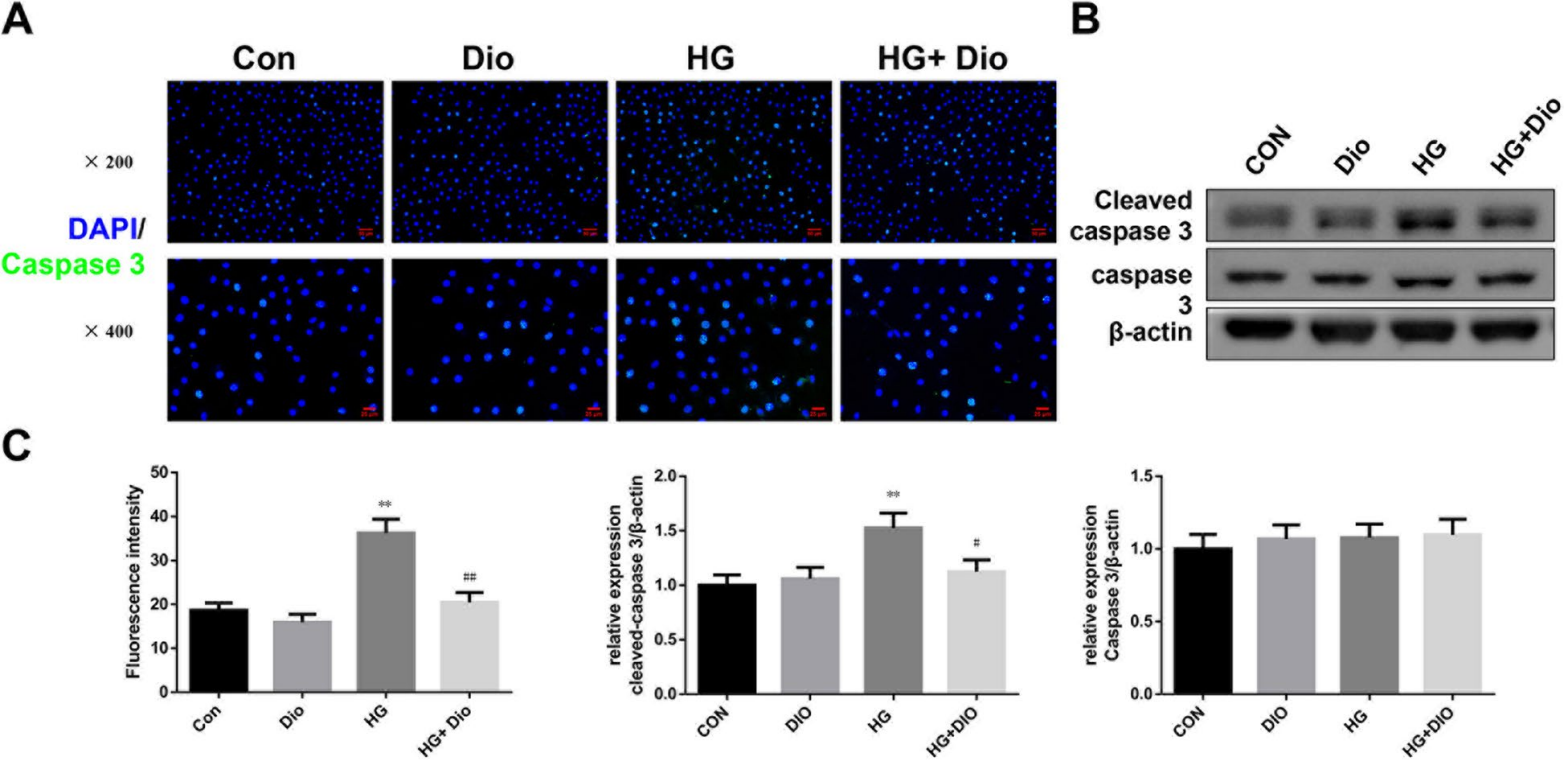
The enhancing effect of high glucose on the expression of Caspase-3 was mitigated by diosmin intervention. **A** Caspase-3 expression was measured using immunofluorescence assay, magnification × 200 (scale bar =50 μm), × 400 (scale bar =25 μm), respectively. **B** Western blot assay for detection the protein expression of cleaved caspase-3 and caspase-3. **C** Statistical analysis for the semi - quantitative of immunofluorescence assay and Western blot assay. The results were expressed as the mean ± standard deviation (SD) of three independent experiments. Compared with the control group, ** *p* < 0.01. Compared with the high glucose group, ## *p* < 0.01

In Fig. 4A, we examined the positive expression of Caspase-3 by immunofluorescence assay and discovered that diosmin alone did not affect the positive expression of Caspase-3 relative to the control group, while the expression of Caspase-3 was enhanced by HG. Moreover, diosmin intervention restrained the positive expression of Caspase-3 induced by HG (Fig. 4A, C). In addition, the positive expression of cleaved Caspase-3 which was examined by western blot got the same results (Fig. 4B, C, *P* < 0.01).

### The diosmin intervention weakened the activation effect of HG on the expressions of CHOP and GRP78

**Fig. 5:**
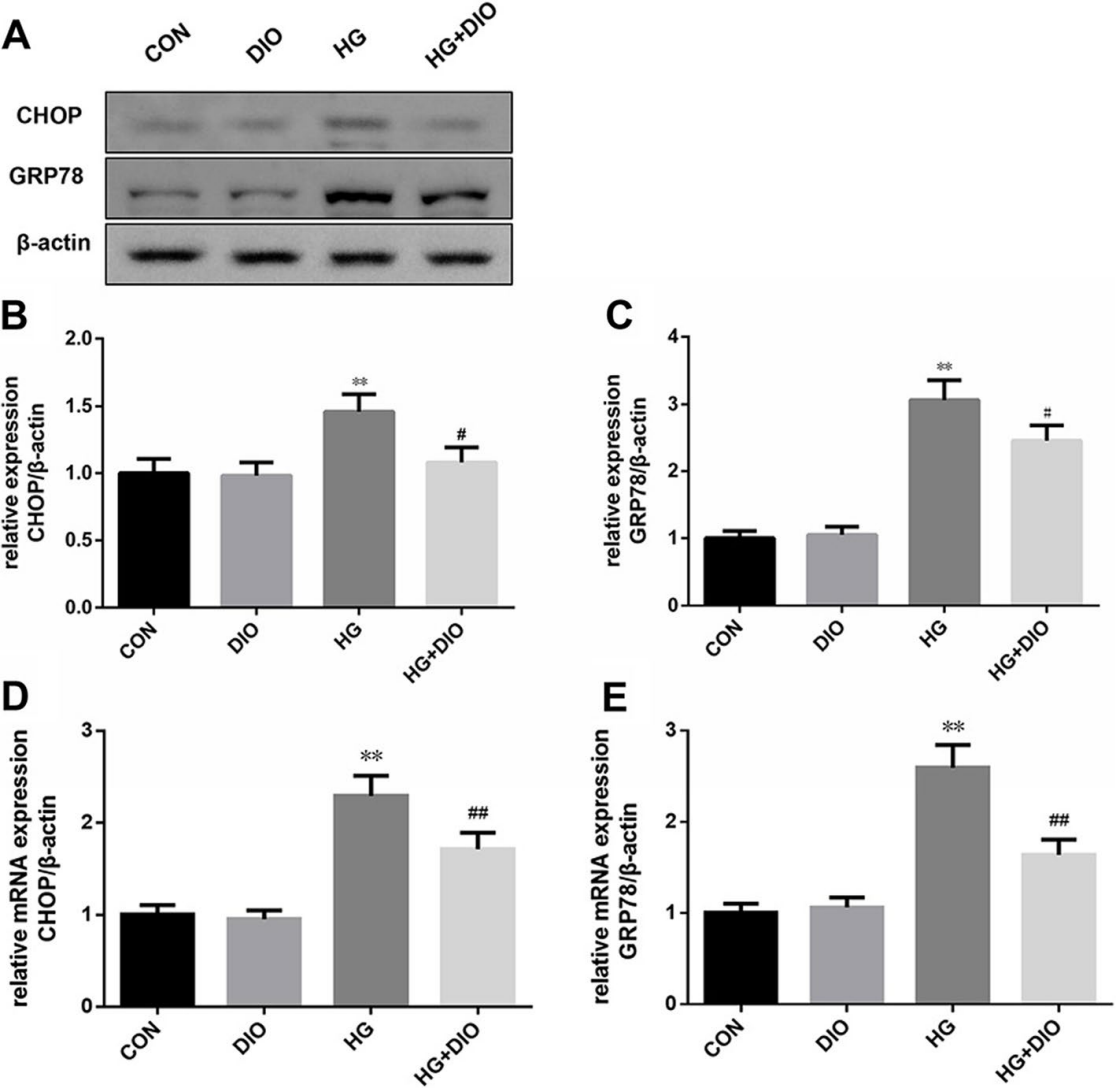
The effect of diosmin on the levels of endoplasmic reticulum stress-related proteins in HK-2 cells induced by high glucose. **A-E**. The protein and mRNA expressions of glucose regulated protein 78 (GRP78) and C/EBP homologous protein (CHOP) in HK-2 cells were detected by western blot and quantitative real-time polymerase chain reaction (qRT-PCR). β-actin was used as a housekeeping gene. The results were expressed as the mean ± standard deviation (SD) of three independent experiments. Compared with the control group, ** *p* < 0.01. Compared with the high glucose group, # *p* < 0.05 and ## *p* < 0.01

ERS plays a pivotal role in the initiation and development of diabetes and DN, thus we examined the expressions of CHOP and GRP78. Diosmin alone did not affect the mRNA and protein levels of CHOP and GRP78, while HG obviously elevated the mRNA and protein levels of CHOP and GRP78 (Fig. 5A-E, *P* < 0.01). Meanwhile, we also discovered that diosmin treatment strongly repressed the mRNA and protein levels of CHOP and GRP78 induced by HG in HK-2 cells (Fig. 5A-E, *P* < 0.05).

### The effect of diosmin on oxidative stress-, inflammation-, apoptosis-, and autophagy-related factors in HG-mediated HK-2 cells

**Fig. 6:**
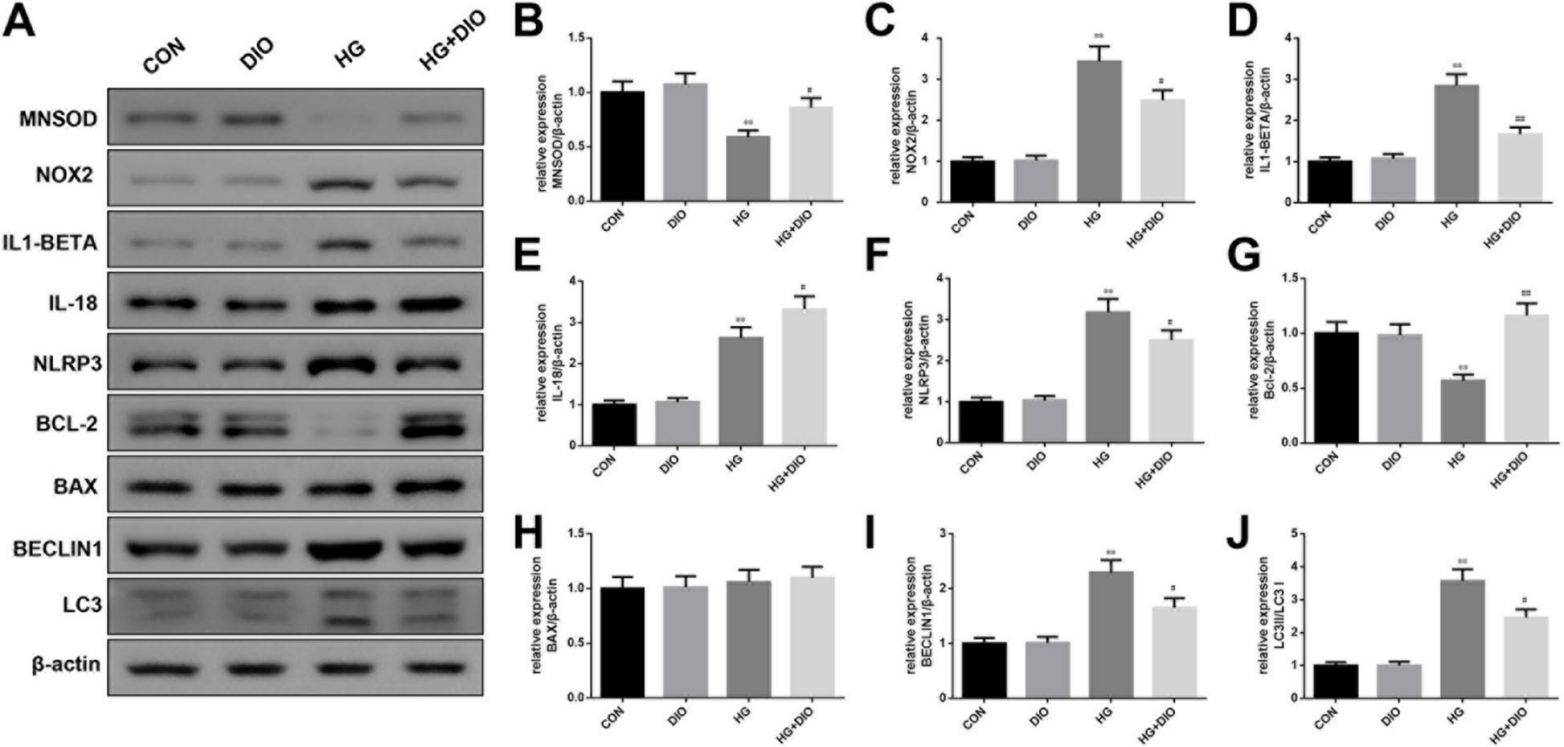
The effect of diosmin on oxidative stress-, inflammation-, apoptosis-, and autophagy-related factors in high glucose-mediated HK-2 cells. **A-J**. The effect of diosmin on oxidative stress-, inflammation-, apoptosis-, and autophagy-related factors in high glucose-mediated HK-2 cells was detected by western blot. β-actin was used as a housekeeping gene. The results were expressed as the mean ± standard deviation (SD) of three independent experiments. Compared with the control group, ** *p* < 0.01. Compared with the high glucose group, # *p* < 0.05 and ## *p* < 0.01

The results in this section clarified that compared with the control group, the protein levels of MNSOD and BCL-2 in the HG group were largely repressed, while the levels of NOX2, IL-1β, IL-18, NLRP3, BECLIN1 as well as the ratio of LC3 II / I in the HG group were largely enhanced (Fig. 6A-J, *P* < 0.01), but there was no evident change in the level of BAX. Nevertheless, the above effects were reversed by co-treatment of HG and diosmin in HK-2 cells (Fig. 6A-J, *P* < 0.05).

### The effect of diosmin on PI3K/AKT/PTEN signal transduction in HG-mediated HK-2 cells

**Fig. 7:**
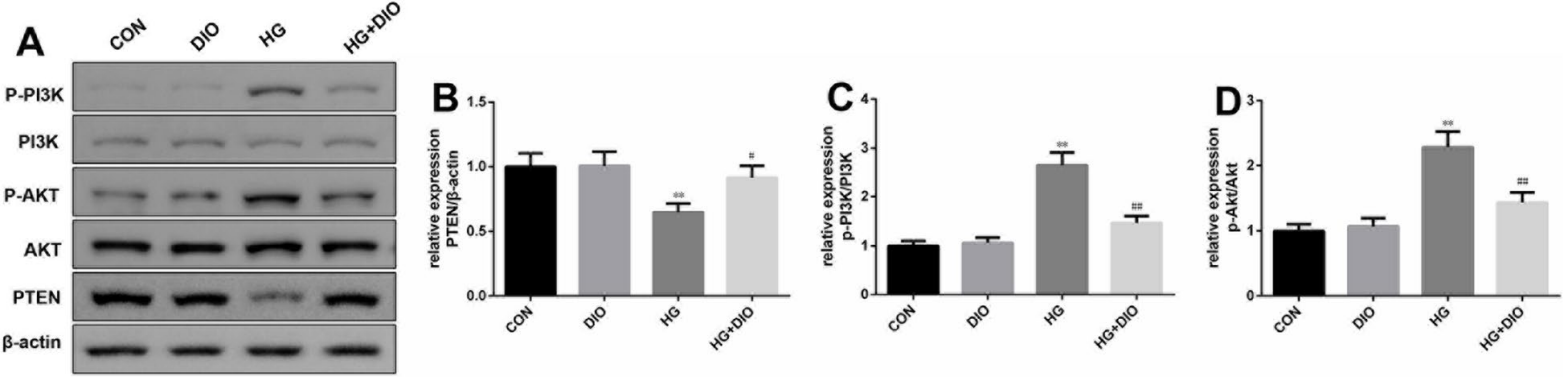
The effect of diosmin on PI3K/AKT/PTEN signal transduction in high glucose-mediated HK-2 cells. **A-D**. The effect of diosmin on phosphatidylinositol 3-kinase/protein kinase-B/ phosphatase and tensin homolog deleted on chromosome 10 pathway-related markers was detected by western blot. β-actin was used as a housekeeping gene. The results were expressed as the mean ± standard deviation (SD) of three independent experiments. Compared with the control group, ** *p* < 0.01. Compared with the high glucose group, # *p* < 0.05 and ## *p* < 0.01

In order to explore whether diosmin alleviated HG-mediated HK-2 cell injury through PI3K/AKT/PTEN pathway, we measured the PI3K/AKT/PTEN pathway-related proteins expression by western blot. There was no evident change in the level of PTEN as well as the ratios of p-PI3K/PI3K and p-AKT/AKT after diosmin treatment alone (Fig. 7A-D). In the HG group, the level of PTEN was restrained and the ratios of p-PI3K/PI3K and p-AKT/AKT were enhanced relative to the control group (Fig. 7A-D, *P* < 0.01). Nevertheless, the regulatory effect of HG on the level of PTEN as well as the ratios of p-PI3K/PI3K and p-AKT/AKT was partially offset by diosmin addition (Fig. 7A-D, *P* < 0.05).

## Discussion

The pathogenesis of DN is multifaceted. In recent years, people have gradually realized the role of stress factors in DN, especially the role of ERS in DN is particularly important [21]. Hyperglycemia is the most important factor leading to the initiation and development of DN, and in vitro studies on hyperglycemia and ERS have shown that hyperglycemia can stimulate the expression of ER-related proteins in renal mesangial cells and ERS-related apoptosis [22]. In this research, the results confirmed that after HG stimulation, the HK-2 cell viability was largely blunted, while the apoptosis rate and Caspase-3 expression were enhanced, which was consistent with the results of Lv et al. [23]. Further, we discovered that diosmin co-incubation with HG-mediated HK-2 cells enhanced the cell vitality but restrained the apoptosis and Caspase-3 expression induced by HG.

Previous studies on the apoptosis pathway of HK-2 cells mainly focused on the mitochondrial internal pathway and the Fas death receptor external pathway, in recent years, it has been manifested that the ERS-mediated apoptosis pathway also played a crucial role in the progression of DN [24]. Endoplasmic reticulum (ER) is a kind of multifunctional organelle which can participate in the regulation of intracellular homeostasis [25]. GRP78 is the main molecular chaperone in the ER and belongs to the HSP70 family [26]. ER is activated in a state of stress accompanied with the elevated expression of GRP78, which is considered to be a landmark protein of ERS activation [27]. Then the activated CHOP is involved in regulating the expression of downstream apoptosis-related genes, including down-regulating the expression of anti-apoptosis gene Bcl-2 and promoting the production of reactive oxygen species [28]. Therefore, the up-regulation of CHOP is usually regarded as a sign of excessive ERS. In this research, we confirmed the activation of the ERS event in HG-stimulated HK-2 cells according to the great increase of MDA content and great reductions of SOD and CAT activities, as well as the repressed MNSOD and BCL-2 levels and the elevated NOX2, CHOP, and GRP78 levels. More importantly, we demonstrated that diosmin addition attenuated the ERS in HG-stimulated HK-2 cells, reversed the regulation of HG on MDA, SOD, CAT, BCL-2, MNSOD, and NOX2 expressions by blocking the CHOP and GRP78 expressions.

On the basis of previous studies, we further studied the expression of inflammation and autophagy-related factors closely related to DN. Autophagy can regulate the inflammatory response that is harmful to cells and avoid damage to the body [29]. Hyperglycemia and glucose metabolism disorders can lead to the increased production of advanced glycation end products, which can activate the oxygen free radicals and NF-κB signaling pathways after binding to receptors, leading to the oxidative stress and inflammation [30]. NLRP3 inflammasomes are currently the focus of research on the induction of inflammatory response. The activation of NLRP3 can lead to the assembly of the NLRP3 inflammasome, this further promotes the self-cleavage of pro-caspase-1 into the activated form of caspase-1, and the activated caspase-1 cleaves pro-IL-1β and pro -IL-18 into IL-1 β and IL-18, which in turn produces an inflammatory cascade [31, 32]. The research in this study clarified that the levels of IL-1β, IL-18, NLRP3, BECLIN1 as well as the ratio of LC3I/LC3II in the HG group cells were largely enhanced. This indicated that HG stimulation induced the inflammation response and cell autophagy in HK-2 cells, which contributed to the ERS and cell injury in simulated DN. We further discovered that the addition of diosmin in HG-HK-2 cells largely blunted the effect of HG on these indicators, which suggested the anti-inflamamtion and autophagy regulation effect of diosmin.

In order to further clarify the exact mechanism by which diosmin alleviates HG-induced ERS in HK-2 cells, the expression of the PTEN/PI3K/AKT pathway-related proteins closely related to the pathogenesis of DN were detected. PTEN is mainly the dephosphorylation of PIP3 downstream of PI3K to PIP2, thereby inhibiting the activity of AKT and ultimately blocking the PI3K/AKT pathway [33]. The latest research suggested that PTEN may be a kidney-protective gene, and overexpressed PTEN can negatively modulate the PI3K/AKT pathway, restrain the AKT activation, improve the phenotype of renal podocytes, and alleviate the renal podocyte damage [34, 35]. The study has also clarified that PTEN expression is down-regulated in HG environments, which can promote renal tubulointerstitial fibrosis by restraining autophagy [36]. Our research found that the level of PTEN was restrained and the ratios of p-PI3K/PI3K and p-AKT/AKT were enhanced in HK-2 cells induced by HG, while which was reversed by co-treatment of diosmin.

In conclusion, our study found that diosmin alleviated the ERS induced by HG in HK-2 cells by blocking the PI3K/AKT pathway. This indicated that diosmin may be used as an potential agent to treat DN. However, this study also has limitations, we will conduct animal experiments to further verify our results in vivo.

## Supplementary Information

**Additional file 1: Supplementary file 1**. The full length membranes images of western blot assay. **Supplementary Figure S1**. Full length membrane of caspase 3 and Cleaved caspase 3 in Fig. 4 of the manuscript. **Supplementary Figure S2**. Full length membrane of β-actin in Fig. 4 of the manuscript. **Supplementary Figure S3**. Full length membrane of CHOP and GRP78 in Fig. 5 of the manuscript. **Supplementary Figure S4**. Full length membrane of β-actin in Fig. 5 of the manuscript. **Supplementary Figure S5**. Full length membrane of MNSOD in Fig. 6 of the manuscript. **Supplementary Figure S6**. Full length membrane of NOX2 in Fig. 6 of the manuscript. **Supplementary Figure S7**. Full length membrane of IL1-BETA and IL-18 in Fig. 6 of the manuscript. **Supplementary Figure S8**. Full length membrane of NLRP3 in Fig. 6 of the manuscript. **Supplementary Figure S9**. Full length membrane of BCL-2 in Fig. 6 of the manuscript. **Supplementary Figure S10**. Full length membrane of BAX in Fig. 6 of the manuscript. **Supplementary Figure S11**. Full length membrane of BECLIN1 in Fig. 6 of the manuscript. **Supplementary Figure S12**. Full length membrane of LC3 in Fig. 6 of the manuscript. **Supplementary Figure S13**. Full length membrane of β-actin in Fig. 6 of the manuscript. **Supplementary Figure S14**. Full length membrane of P-PI3K in Fig. 7 of the manuscript. **Supplementary Figure S15**. Full length membrane of PI3K in Fig. 7 of the manuscript. **Supplementary Figure S16**. Full length membrane of P-AKT in Fig. 7 of the manuscript. **Supplementary Figure S17**. Full length membrane of AKT in Fig. 7 of the manuscript. **Supplementary Figure S18**. Full length membrane of PTEN in Fig. 7 of the manuscript. **Supplementary Figure S19**. Full length membrane of β-actin in Fig. 7 of the manuscript.

## Data Availability

The datasets used and/or analysed during the current study are available from the corresponding author on reasonable request.

## References

[1] Sharaf El Din UAA, Salem MM, Abdulazim DO (2017). Diabetic nephropathy: time to withhold development and progression - a review. J Adv Res.

[2] Hoshino J (2018). A new pathological scoring system by the Japanese classification to predict renal outcome in diabetic nephropathy. PLoS One.

[3] Vallon V, Thomson SC (2012). Renal function in diabetic disease models: the tubular system in the pathophysiology of the diabetic kidney. Annu Rev Physiol.

[4] Zhu Y (2016). RIPK3-mediated Necroptosis and apoptosis contributes to renal tubular cell progressive loss and chronic kidney disease progression in rats. PLoS One.

[5] Arora MK, Singh UK (2013). Molecular mechanisms in the pathogenesis of diabetic nephropathy: an update. Vasc Pharmacol.

[6] Kanwar YS (2011). A glimpse of various pathogenetic mechanisms of diabetic nephropathy. Annu Rev Pathol.

[7] El-Fawal R, El Fayoumi HM, Mahmoud MF (2018). Diosmin and crocin alleviate nephropathy in metabolic syndrome rat model: effect on oxidative stress and low grade inflammation. Biomed Pharmacother.

[8] Carballo-Villalobos AI (2018). Central and peripheral anti-hyperalgesic effects of diosmin in a neuropathic pain model in rats. Biomed Pharmacother.

[9] Mirshekar MA (2017). Diosmin improved cognitive deficit and amplified brain electrical activity in the rat model of traumatic brain injury. Biomed Pharmacother.

[10] Ahmed S (2016). Diosmin modulates the NF-kB signal transduction pathways and Downregulation of various oxidative stress markers in Alloxan-induced diabetic nephropathy. Inflammation.

[11] Urios P (2014). A flavonoid fraction purified from Rutaceae aurantiae (Daflon(R)) inhibiting AGE formation, reduces urinary albumin clearance and corrects hypoalbuminemia in normotensive and hypertensive diabetic rats. Diabetes Res Clin Pract.

[12] Bravo R (2013). Endoplasmic reticulum and the unfolded protein response: dynamics and metabolic integration. Int Rev Cell Mol Biol.

[13] Jaikumkao K (2018). Dapagliflozin, a sodium-glucose co-transporter-2 inhibitor, slows the progression of renal complications through the suppression of renal inflammation, endoplasmic reticulum stress and apoptosis in prediabetic rats. Diabetes Obes Metab.

[14] Wang ZS (2015). Astragaloside IV attenuates proteinuria in streptozotocin-induced diabetic nephropathy via the inhibition of endoplasmic reticulum stress. BMC Nephrol.

[15] Song Y (2020). Mangiferin alleviates renal interstitial fibrosis in Streptozotocin-induced diabetic mice through regulating the PTEN/PI3K/Akt signaling pathway. J Diabetes Res.

[16] Chen X (2013). Ghrelin induces cell migration through GHSR1a-mediated PI3K/Akt/eNOS/NO signaling pathway in endothelial progenitor cells. Metabolism.

[17] Kasinath BS (2008). Novel mechanisms of protein synthesis in diabetic nephropathy--role of mRNA translation. Rev Endocr Metab Disord.

[18] Liu WY (2017). The benefits of the Citrus flavonoid Diosmin on human retinal pigment epithelial cells under high-glucose conditions. Molecules.

[19] Ju Y (2019). Protective effects of Astragaloside IV on endoplasmic reticulum stress-induced renal tubular epithelial cells apoptosis in type 2 diabetic nephropathy rats. Biomed Pharmacother.

[20] Schmittgen TD, Livak KJ (2008). Analyzing real-time PCR data by the comparative C(T) method. Nat Protoc.

[21] Fan Y (2017). The role of endoplasmic reticulum stress in diabetic nephropathy. Curr Diab Rep.

[22] Yao F (2015). Fatty acid-binding protein 4 mediates apoptosis via endoplasmic reticulum stress in mesangial cells of diabetic nephropathy. Mol Cell Endocrinol.

[23] Lv L (2019). Arbutin protects HK-2 cells against high glucose-induced apoptosis and autophagy by up-regulating microRNA-27a. Artif Cells Nanomed Biotechnol.

[24] Sun XY (2016). Valproate attenuates diabetic nephropathy through inhibition of endoplasmic reticulum stress-induced apoptosis. Mol Med Rep.

[25] Fu XL, Gao DS (2014). Endoplasmic reticulum proteins quality control and the unfolded protein response: the regulative mechanism of organisms against stress injuries. Biofactors.

[26] McGuckin MA (2010). ER stress and the unfolded protein response in intestinal inflammation. Am J Physiol Gastrointest Liver Physiol.

[27] Meir O (2010). C/EBP-beta regulates endoplasmic reticulum stress-triggered cell death in mouse and human models. PLoS One.

[28] Tabas I, Ron D (2011). Integrating the mechanisms of apoptosis induced by endoplasmic reticulum stress. Nat Cell Biol.

[29] Ko JH (2017). Rapamycin regulates macrophage activation by inhibiting NLRP3 inflammasome-p38 MAPK-NFκB pathways in autophagy- and p62-dependent manners. Oncotarget.

[30] Wang Z (2019). Protective effects of pyrroloquinoline quinine against oxidative stress-induced cellular senescence and inflammation in human renal tubular epithelial cells via Keap1/Nrf2 signaling pathway. Int Immunopharmacol.

[31] Franchi L (2009). The inflammasome: a caspase-1-activation platform that regulates immune responses and disease pathogenesis. Nat Immunol.

[32] Kang KS (2007). Preventive effect of 20(S)-ginsenoside Rg3 against lipopolysaccharide-induced hepatic and renal injury in rats. Free Radic Res.

[33] Zhang J (2018). Surface chemistry induces mitochondria-mediated apoptosis of breast cancer cells via PTEN/PI3K/AKT signaling pathway. Biochim Biophys Acta Mol Cell Res.

[34] Wang H (2018). Podocyte-specific knockin of PTEN protects kidney from hyperglycemia. Am J Physiol Renal Physiol.

[35] Li XY (2017). Triptolide restores autophagy to alleviate diabetic renal fibrosis through the miR-141-3p/PTEN/Akt/mTOR pathway. Mol Ther Nucleic Acids.

[36] Liu X (2018). Notch1 regulates PTEN expression to exacerbate renal tubulointerstitial fibrosis in diabetic nephropathy by inhibiting autophagy via interactions with Hes1. Biochem Biophys Res Commun.

