# Supplementary material for: Diosmin mitigates high glucose-induced endoplasmic reticulum stress through PI3K/AKT pathway in HK-2 cells
Source: BMC Complement Med Ther. 2022 Apr 27;22:116. doi: 10.1186/s12906-022-03597-y (PMC9044681; doi:10.1186/s12906-022-03597-y)
Supplement: Supplementary file 1 — Additional file 1: Supplementary file 1. The full length membranes images of western blot assay. Supplementary Figure S1. Full length membrane of caspase 3 and Cleaved caspase 3 in Fig. 4 of the manuscript. Supplementary Figure S2. Full length membrane of β-actin in Fig. 4 of the manuscript. Supplementary Figure S3. Full length membrane of CHOP and GRP78 in Fig. 5 of the manuscript. Supplementary Figure S4. Full length membrane of β-actin in Fig. 5 of the manuscript. Supplementary Figure S5. Full length membrane of MNSOD in Fig. 6 of the manuscript. Supplementary Figure S6. Full length membrane of NOX2 in Fig. 6 of the manuscript. Supplementary Figure S7. Full length membrane of IL1-BETA and IL-18 in Fig. 6 of the manuscript. Supplementary Figure S8. Full length membrane of NLRP3 in Fig. 6 of the manuscript. Supplementary Figure S9. Full length membrane of BCL-2 in Fig. 6 of the manuscript. Supplementary Figure S10. Full length membrane of BAX in Fig. 6 of the manuscript. Supplementary Figure S11. Full length membrane of BECLIN1 in Fig. 6 of the manuscript. Supplementary Figure S12. Full length membrane of LC3 in Fig. 6 of the manuscript. Supplementary Figure S13. Full length membrane of β-actin in Fig. 6 of the manuscript. Supplementary Figure S14. Full length membrane of P-PI3K in Fig. 7 of the manuscript. Supplementary Figure S15. Full length membrane of PI3K in Fig. 7 of the manuscript. Supplementary Figure S16. Full length membrane of P-AKT in Fig. 7 of the manuscript. Supplementary Figure S17. Full length membrane of AKT in Fig. 7 of the manuscript. Supplementary Figure S18. Full length membrane of PTEN in Fig. 7 of the manuscript. Supplementary Figure S19. Full length membrane of β-actin in Fig. 7 of the manuscript. [file 12906_2022_3597_MOESM1_ESM.pdf]

Supplementary file 1. The full length membranes images of western blot assay

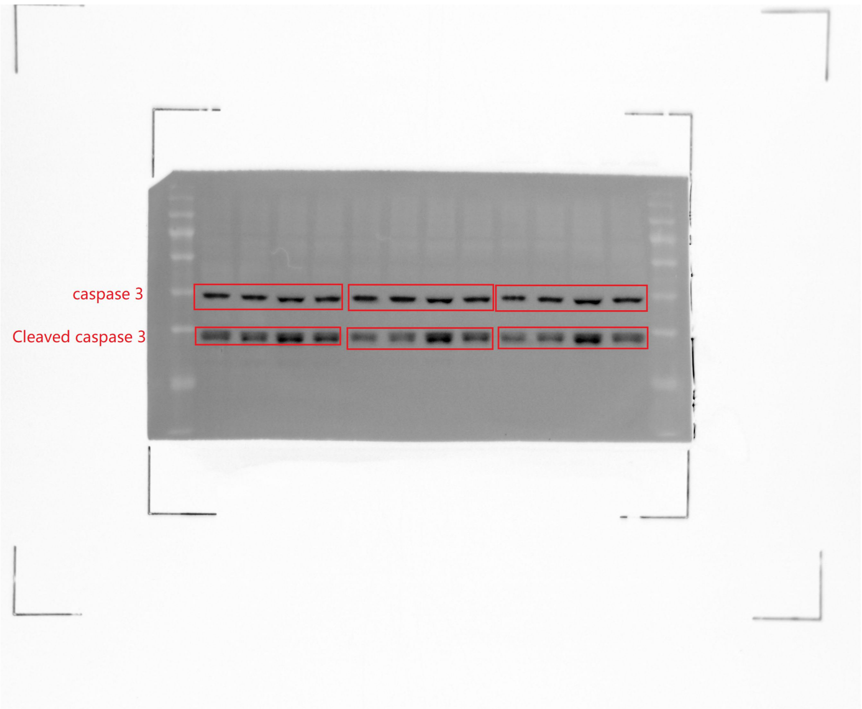

Supplementary figure S1. Full length membrane of caspase 3 and Cleaved caspase 3 in Figure 4 of the manuscript.

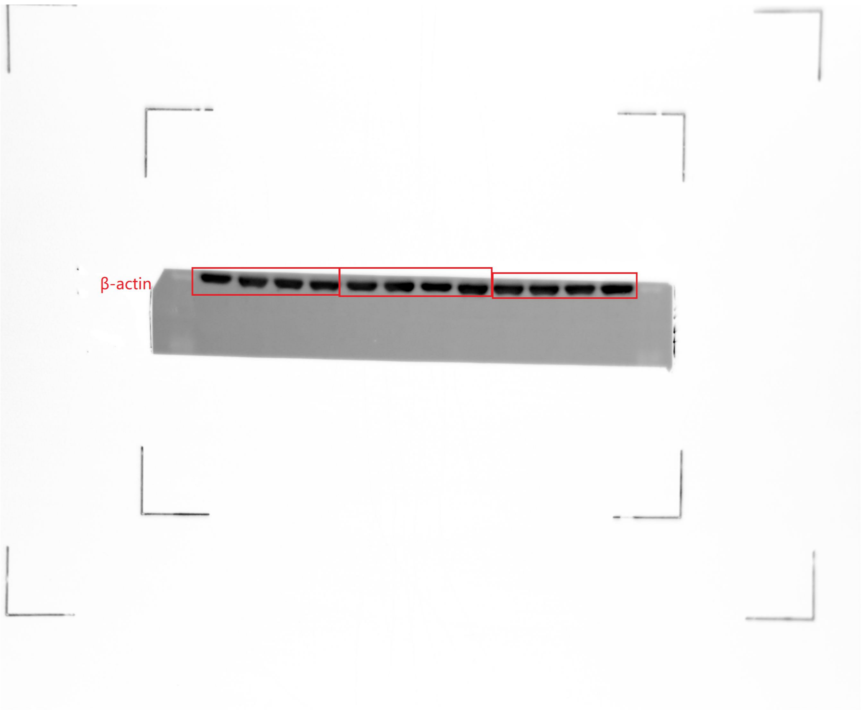

Supplementary figure S2. Full length membrane of β-actin in Figure 4 of the manuscript.

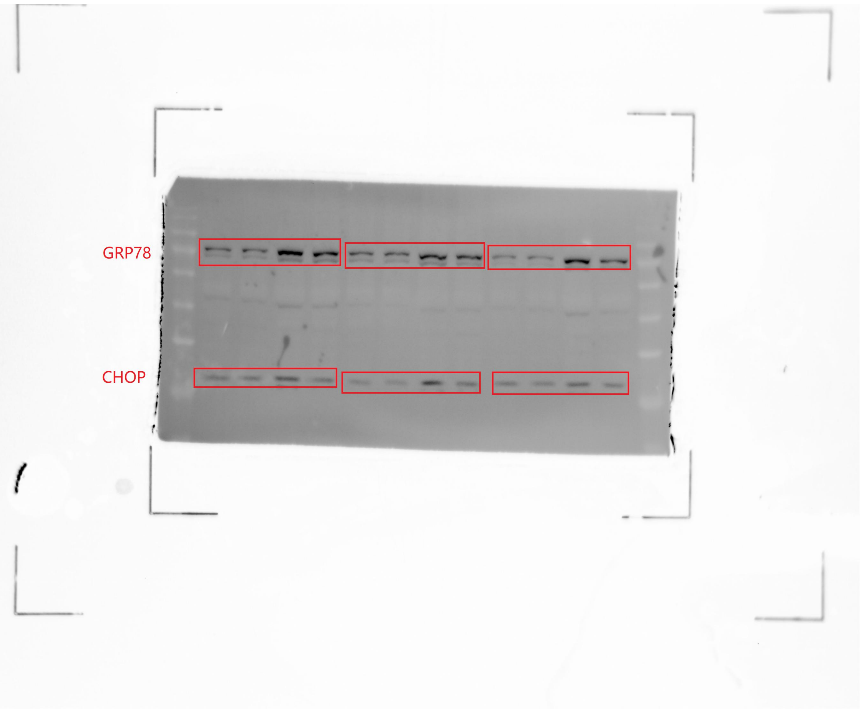

Supplementary figure S3. Full length membrane of CHOP and GRP78 in Figure 5 of the manuscript.

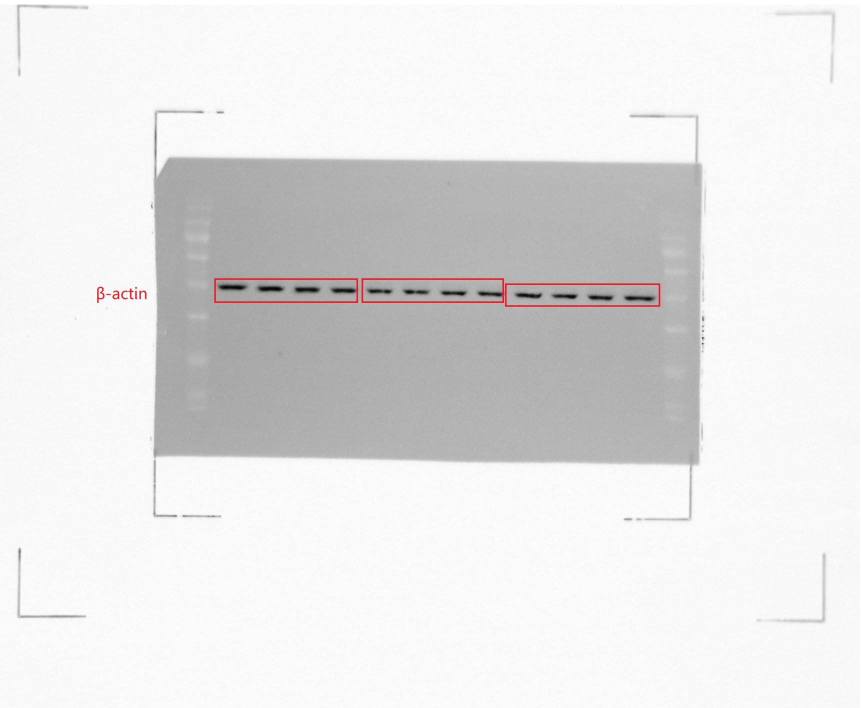

Supplementary figure S4. Full length membrane of β-actin in Figure 5 of the manuscript.

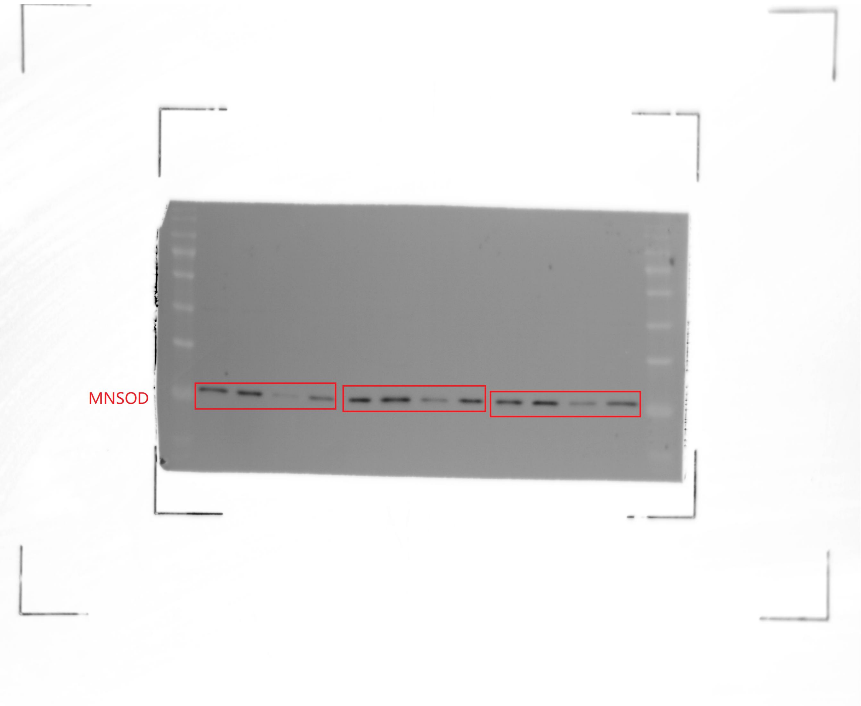

Supplementary figure S5. Full length membrane of MNSOD in Figure 6 of the manuscript.

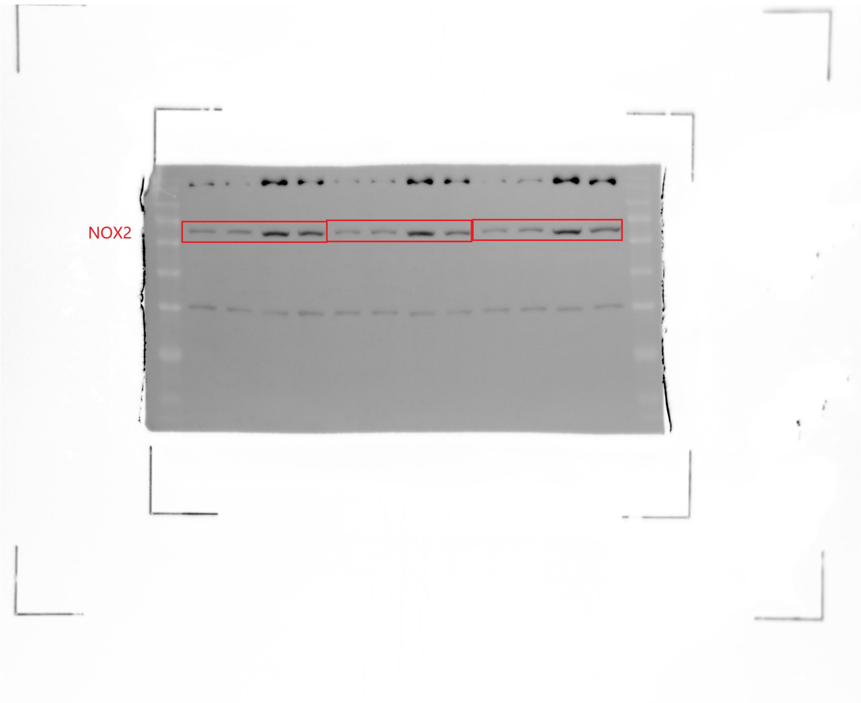

Supplementary figure S6. Full length membrane of NOX2 in Figure 6 of the manuscript.

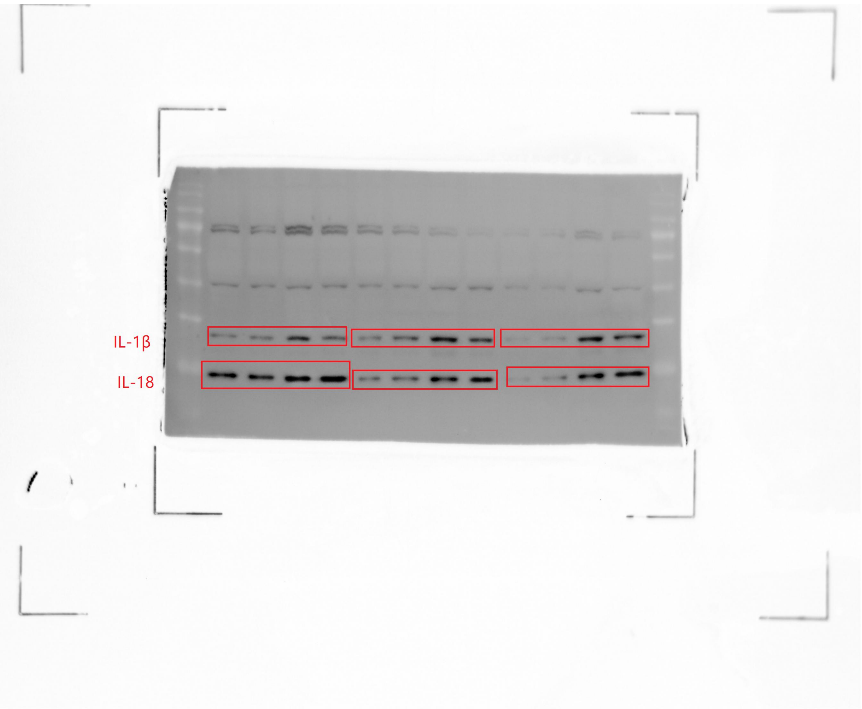

Supplementary figure S7. Full length membrane of IL1-BETA and IL-18 in Figure 6 of the manuscript.

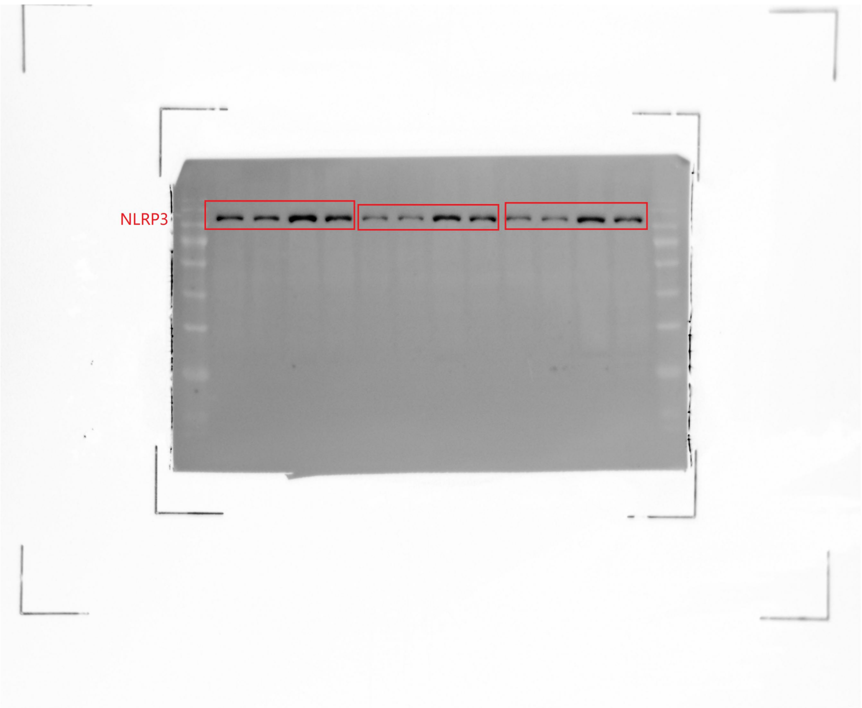

Supplementary figure S8. Full length membrane of NLRP3 in Figure 6 of the manuscript.

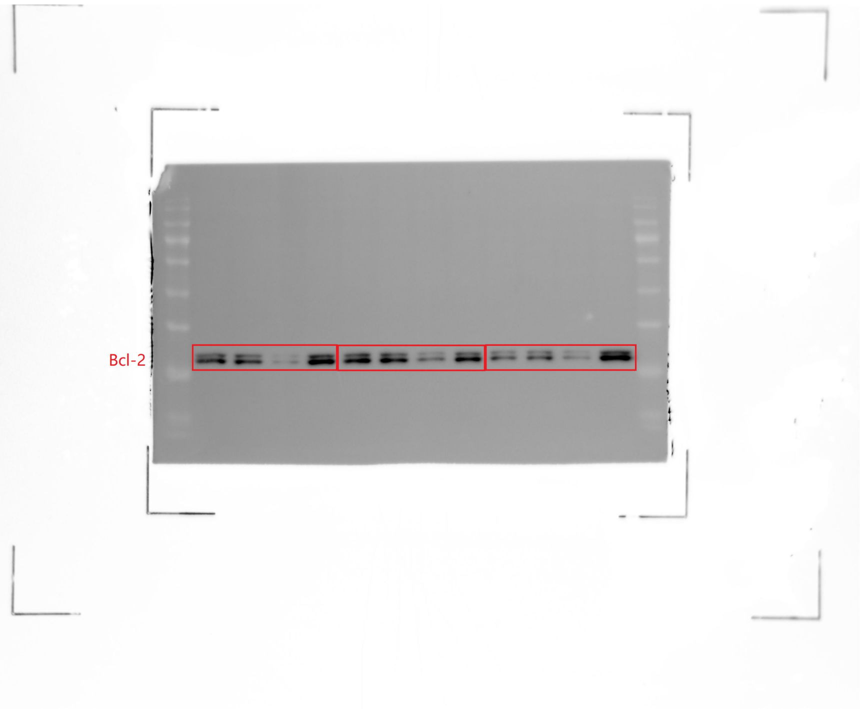

Supplementary figure S9. Full length membrane of BCL-2 in Figure 6 of the manuscript.

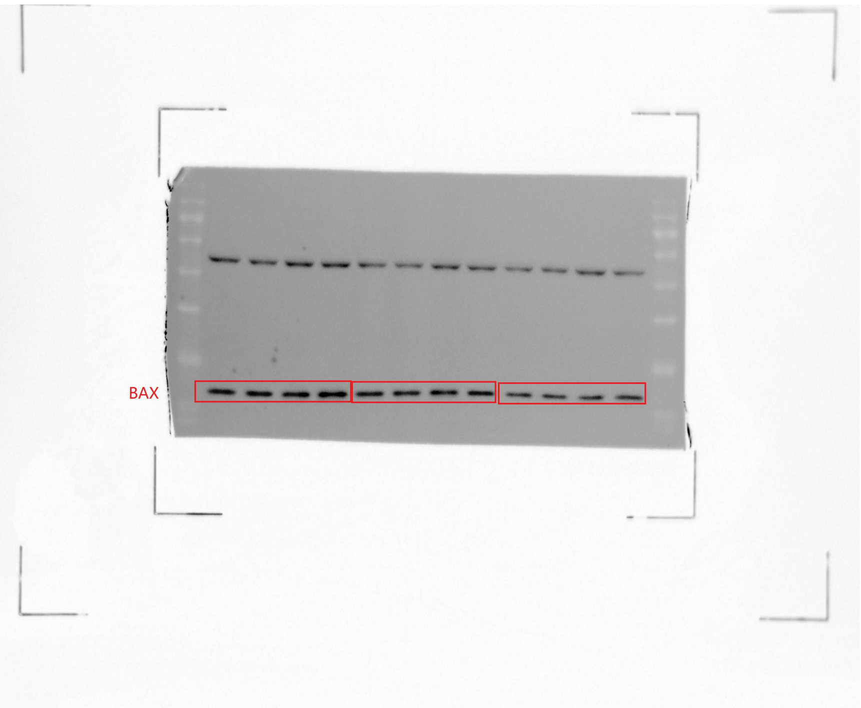

Supplementary figure S10. Full length membrane of BAX in Figure 6 of the manuscript.

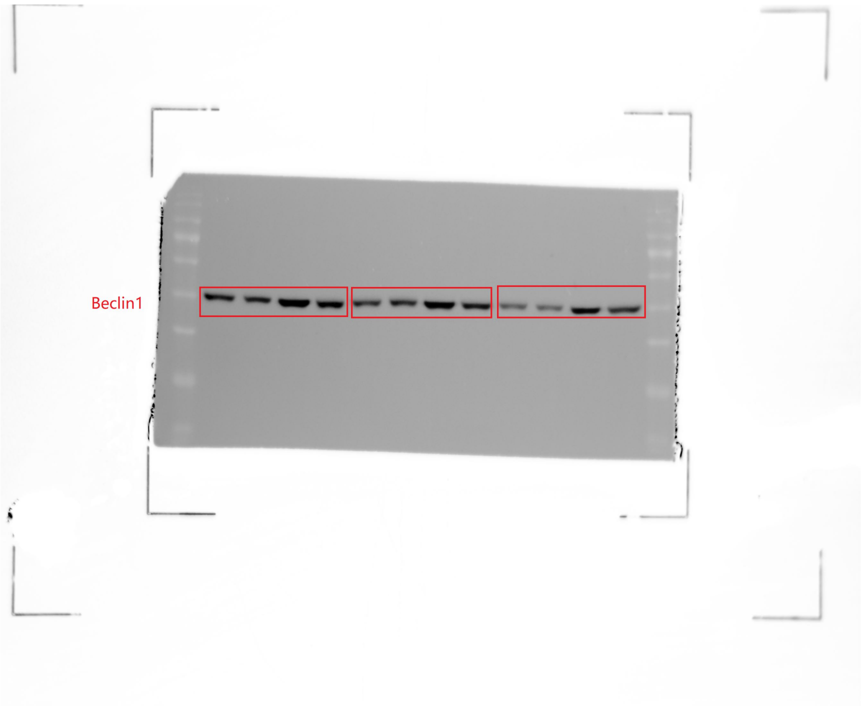

Supplementary figure S11. Full length membrane of BECLIN1 in Figure 6 of the manuscript.

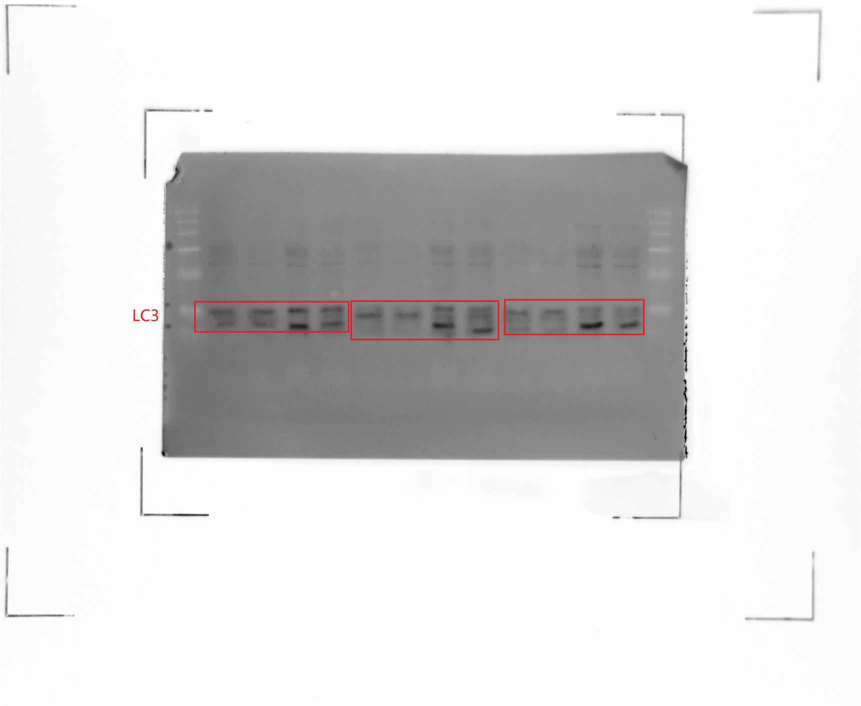

Supplementary figure S12. Full length membrane of LC3 in Figure 6 of the manuscript.

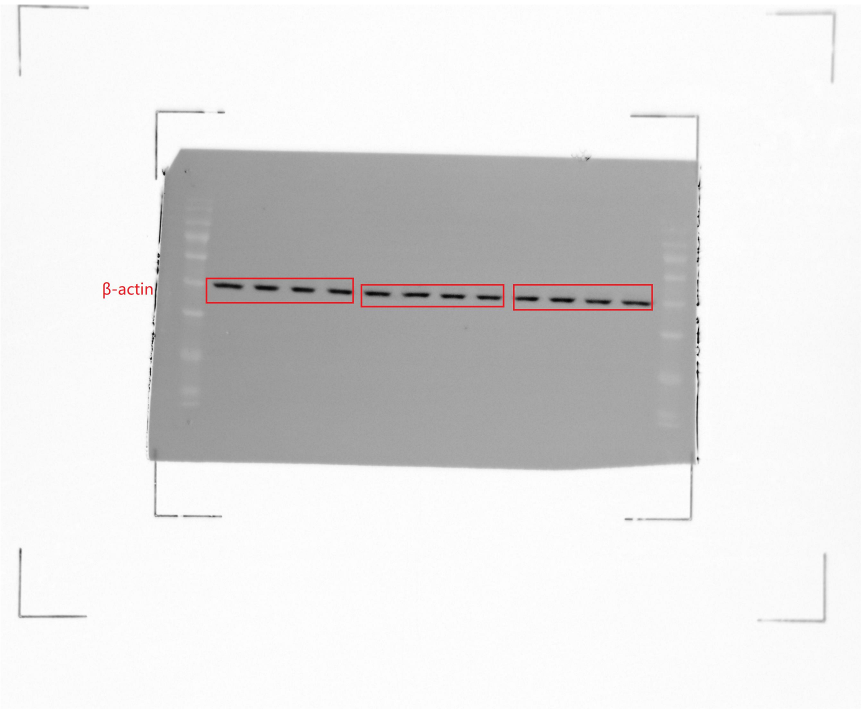

Supplementary figure S13. Full length membrane of β-actin in Figure 6 of the manuscript.

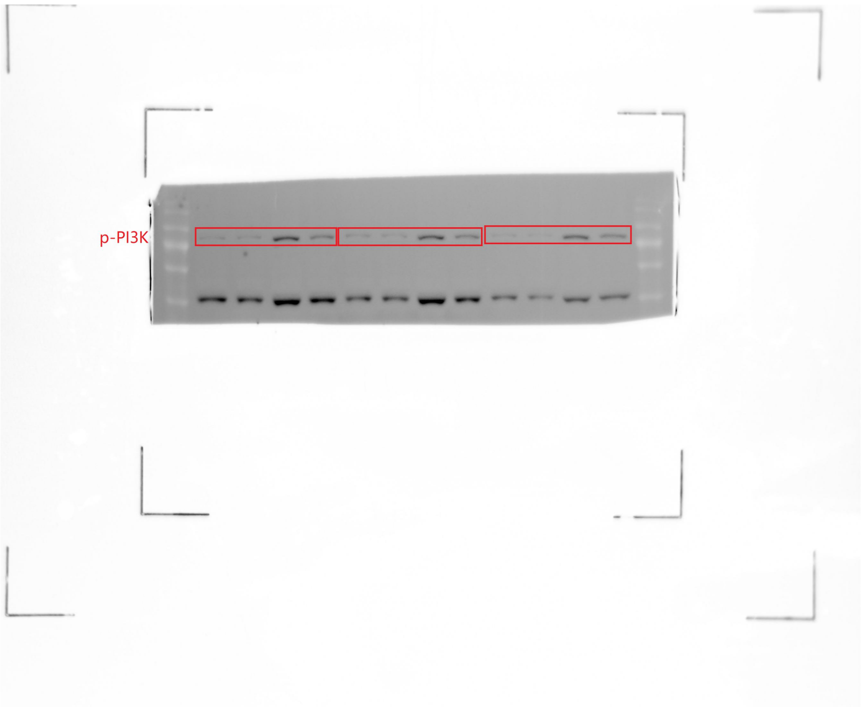

Supplementary figure S14. Full length membrane of P-PI3K in Figure 7 of the manuscript.

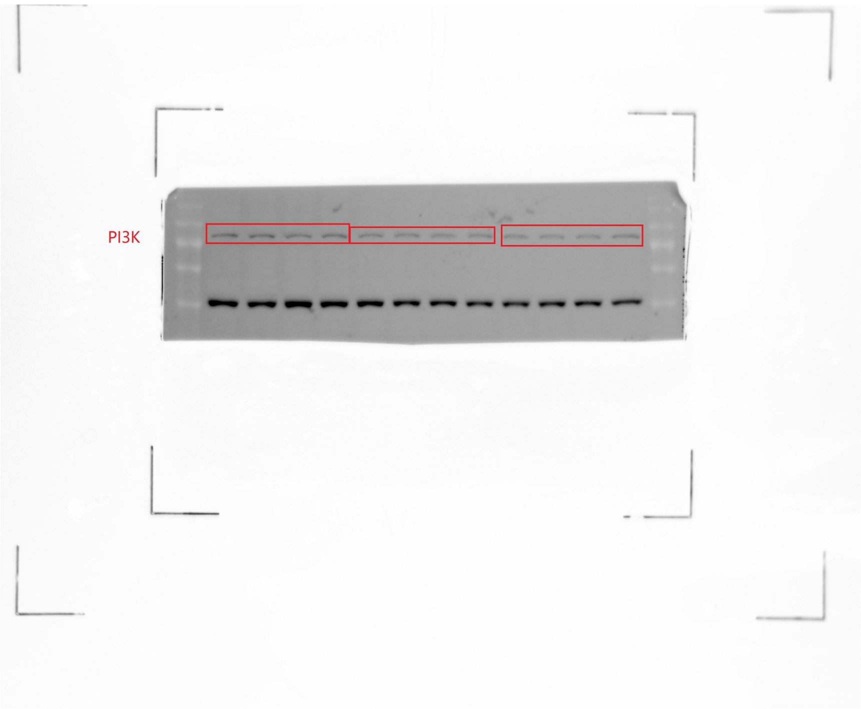

Supplementary figure S15. Full length membrane of PI3K in Figure 7 of the manuscript.

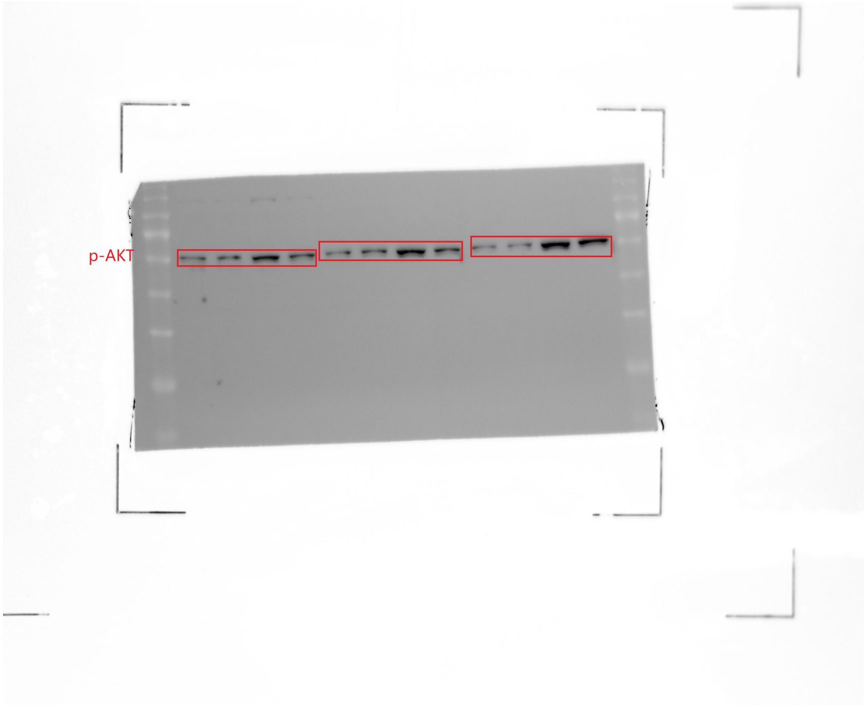

Supplementary figure S16. Full length membrane of P-AKT in Figure 7 of the manuscript.

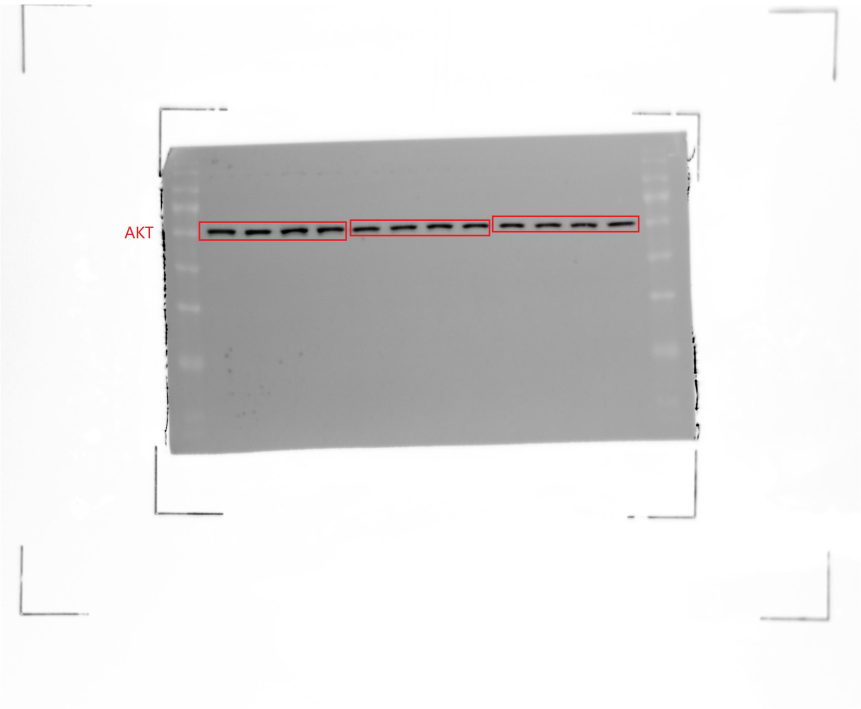

Supplementary figure S17. Full length membrane of AKT in Figure 7 of the manuscript.

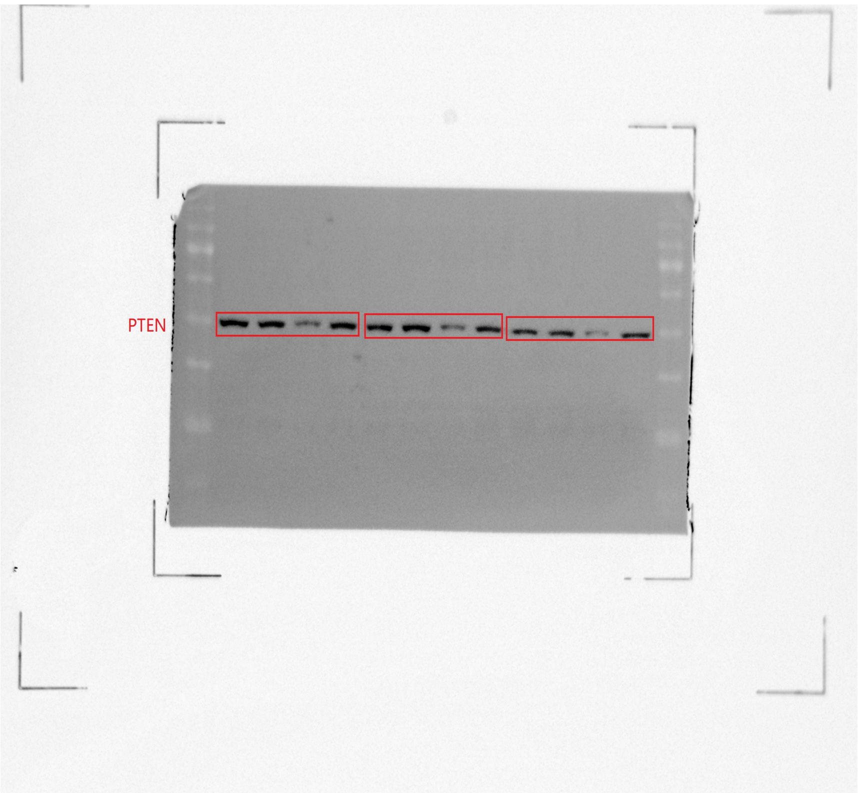

Supplementary figure S18. Full length membrane of PTEN in Figure 7 of the manuscript.

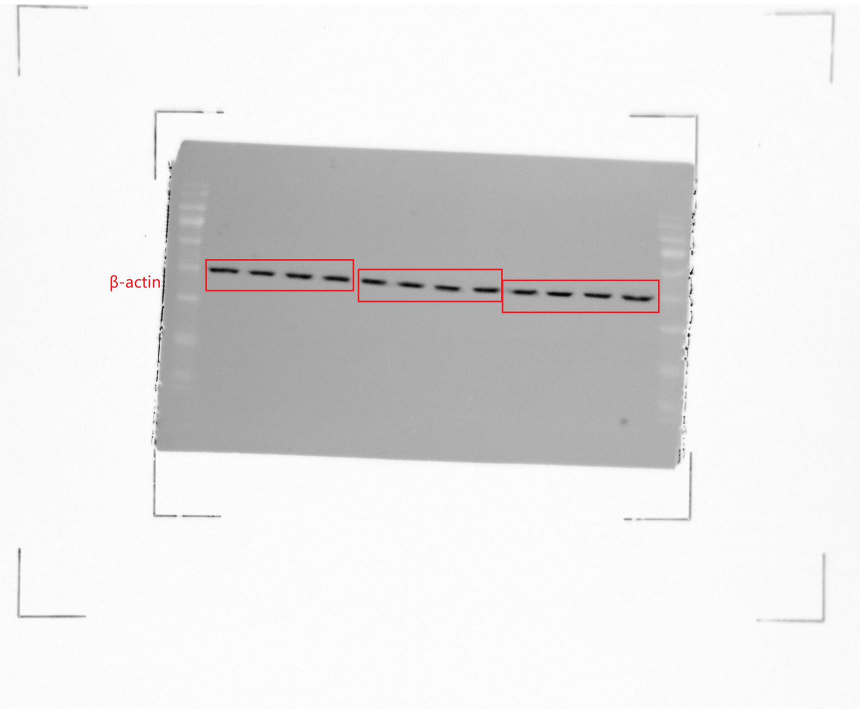

Supplementary figure S19. Full length membrane of β-actin in Figure 7 of the manuscript.
